# White-Matter Structural Connectivity in Relation to Humor Styles: An Exploratory Study

**DOI:** 10.3389/fpsyg.2018.01654

**Published:** 2018-09-04

**Authors:** Ching-Lin Wu, Suyu Zhong, Yu-Chen Chan, Hsueh-Chih Chen, Yong He

**Affiliations:** ^1^Program of Learning Sciences, National Taiwan Normal University, Taipei, Taiwan; ^2^Institute for Research Excellence in Learning Sciences, National Taiwan Normal University, Taipei, Taiwan; ^3^State Key Laboratory of Cognitive Neuroscience and Learning, Beijing Normal University, Beijing, China; ^4^IDG/McGovern Institute for Brain Research, Beijing Normal University, Beijing, China; ^5^Department of Educational Psychology and Counseling, National Tsing Hua University, Hsinchu, Taiwan; ^6^Chinese Language and Technology Center, National Taiwan Normal University, Taipei, Taiwan; ^7^Department of Educational Psychology and Counseling, National Taiwan Normal University, Taipei, Taiwan; ^8^Beijing Key Laboratory of Brain Imaging and Connectomics, Beijing Normal University, Beijing, China

**Keywords:** humor styles, white matter, graph theory, diffusion tensor imaging, human connectome

## Abstract

To investigate the potential relationship between white matter (WM) microstructure and humor styles, diffusion tensor images of brain WM and humor style tendencies were obtained from thirty healthy adults. Using connectivity efficiency measures from graph theoretical analysis and controlling for the influence of gender, age, educational level, and the big five personality traits, we preliminarily examined the prediction of humor styles from brain network efficiency. The results showed that the local efficiency within particular brain networks positively predicted a self-enhancing humor style and negatively predicted an aggressive humor style. The node efficiency of the left superior temporal gyrus distinguished the benevolent or hostile way that individuals coped with interpersonal embarrassment. These findings from this exploratory study support the hypothesis that WM structure influences humor styles, and provide the initial evidence and implications regarding the relationship between biological mechanisms and mental health for future research.

## Introduction

Humor is an advanced function of cognition and is exclusive to humans; it improves social contact and helps people cope with the pressures of life (Long and Graesser, 1988; Lefcourt, 2001). However, humor is a complex concept (Martin, 2001); if people use humor well, it increases the quality of interpersonal situations and mental health, whereas if people abuse humor (e.g., harm or mock others), it can damage their personal social life (Kuiper et al., 2004). Past studies have taken humor to be a positive trait but have underemphasized its harmful sides. Martin et al. (2003) classified humor content into “towards self/towards others" and “kind-hearted/malicious" and claimed that there are four humor styles. This article discusses the negative influence of humor on individuals’ psychological well-being and interpersonal relationships and provides a comprehensive theoretical structure of humor. The four humor styles included affiliative humor, which improved harmonious social interaction; self-enhancing humor, which maintained mental health; aggressive humor, which led to malicious responses; and self-defeating humor, which was harmful to psychological adjustment. People who favored affiliative humor and self-enhancing humor had personalities with higher levels of openness, extraversion, agreeableness, and self-esteem. In particular, people with an affiliative humor style had higher conscientiousness, whereas people with a self-enhancing humor style had lower neuroticism. People who preferred aggressive humor and self-defeating humor had lower levels of conscientiousness but higher levels of neuroticism. Specifically, people with an aggressive humor style had lower levels of agreeableness and openness, whereas people with a self-defeating humor style had higher levels of openness (Greengross et al., 2011; Liu, 2012). In sum, personal humor style was significantly related to personality, self-esteem, and aggressive behaviors (Martin et al., 2003; Stieger et al., 2011; Liu, 2012). However, the influence of neurocognitive factors on personal humor styles remains unknown.

Recently, researchers have had an increasing interest in the neurocognitive correlates of humor (Chan et al., 2012, 2013). However, research on the neural mechanisms behind people’s preference of humor styles remains limited. Most of the studies on this topic have focused on the brain mechanisms by which people understand and enjoy humor material. For instance, humor comprehension was correlated with the activation of the cortex in the frontal and temporal lobes (Goel and Dolan, 2001), and humor appreciation was related to the activation of the subcortex in the mesolimbic system (Mobbs et al., 2005). Chan et al. (2012, 2013) integrated the incongruity-resolution theory (Suls, 1972) and the comprehension-elaboration theory (Wyer and Collins, 1992), analyzed the comprehension and appreciation of humor, and divided the humor process into three stages based on the activation of corresponding brain regions. Using fMRI, these authors showed that during the stage of incongruity detection, the corresponding regions were the right middle temporal gyrus and right medial frontal gyrus; during the stage of incongruity resolution, the corresponding regions were the left superior frontal gyrus and left inferior parietal lobule; and during the stage of elaboration, the corresponding regions were the left ventromedial prefrontal cortex (PFC), bilateral amygdala, and bilateral parahippocampal gyri. Recently, research using a structural brain perspective to study personality and brain structures has become increasingly common, and some studies have investigated the neural relationship between psychological traits (such as personality and creativity) and the diffusion tensor imaging (DTI) of white matter (WM) (Takeuchi et al., 2010; Xu and Potenza, 2012). White matter plays a key role in regulating brain communication and functional integrity (Gong et al., 2009). Therefore, we can acquire more knowledge of the neural structures of humor style preference by investigating the relationship between WM structures and humor styles.

Humor styles and personality are significantly correlated (Martin et al., 2003; Liu, 2012), and a previous study has indicated that the fractional anisotropy (FA) of WM structure is negatively correlated to neuroticism (Bjørnebekk et al., 2013) but positively correlated to openness, agreeableness, extraversion, and aggressive behaviors (Hoptman et al., 2002; Gurrera et al., 2007; Xu and Potenza, 2012). These studies suggest that there is a possible connection between humor styles and WM structure. Additionally, structural brain imaging studies have found a close link between the aforementioned traits and regional activation. Neuroticism was positively correlated to the activation of the anterior cingulate as well as to that of the dorsomedial PFC (Hooker et al., 2008; Harenski et al., 2009; Xu and Potenza, 2012). Openness and extraversion were positively correlated to the interconnection between the parietal lobe, temporal lobe, and dmPFC (Mobbs et al., 2005; Haas et al., 2006; Kitamura et al., 2016), and openness was positively correlated to the communication between cerebral hemispheres (i.e., corpus callosum) (Xu and Potenza, 2012). Agreeableness was positively correlated to the volume of gray matter in the posterior cingulate but negatively correlated to the volume of gray matter in the superior temporal gyrus (DeYoung et al., 2010). Moreover, aggressive tendencies and the activation of the PFC and ACC had positive correlations (Denson et al., 2009), and affiliative motivation was more active in right brain regions (Kuhl and Kazén, 2008). In summary, positive traits were related to activation in the right parietal, temporal, and frontal lobes, but hostile traits were related to activation in the anterior cingulate and frontal lobes.

Furthermore, humor styles include the awareness of the emotions of oneself or others (Martin et al., 2003) and are closely related to self-evaluation (Dozois et al., 2009), empathy (Hampes, 2001, 2010), and theory of mind (Samson et al., 2013). Some brain imaging studies have shown that the self-awareness of emotions and traits was related to the activation of the mPFC, temporal lobe, and PCC (Knutson et al., 2003, 2005; Ochsner et al., 2004). Empathy was related to the activation of the superior and inferior frontal gyri, precuneus, and middle temporal gyrus (Farrow et al., 2001; Mahy et al., 2014), whereas self-other awareness and distinction were related to activation in the mPFC, inferior parietal lobule, and right temporal–parietal junction (Singer and Lamm, 2009). Particularly, the left cerebral hemisphere was the key to self- or other-processing (Turk et al., 2002; Denny et al., 2012), and theory of mind was related to the activation of the middle frontal gyrus, cuneus, and superior temporal gyrus (Vöellm et al., 2006; Schurz et al., 2014). Accordingly, self-awareness is highly relevant to the frontal lobe, temporal lobe, and PCC in the left cerebral hemisphere.

The present study investigated the relationship between humor styles and brain WM by using the DTI technique. DTI analyzes the diffusion of water in brain tissue and provides the mean diffusivity (MD), FA and main direction of diffusivities of water molecules. Additionally, in combination with tractography, researchers acquired structural brain images of brain tissue and neural pathways (Sporns et al., 2004; Achard et al., 2006; Hagmann et al., 2008; van den Heuvel et al., 2008; Gong et al., 2009). Recently, researchers have used graph theoretical analysis to build the network structure of WM according to the FA between brain regions and nerve fiber numbers (Bullmore and Sporns, 2009; He et al., 2009) and used the clustering coefficient (*Cp*) and the characteristic path length (*Lp*) to represent the cluster levels and connectivity efficiency between brain network regions. Other studies have further described the relationships between individual characteristics (e.g., gender, age, and IQ) and relevant attributes of brain network structures (Sporns et al., 2005; van den Heuvel et al., 2009). In these comprehensive investigations, researchers determined the correlation between the connectivity efficiency of the brain network and personal traits.

Several brain imaging studies have examined the neural correlates of processing humor (Chan et al., 2012, 2013), but few studies have studied the relationship between brain structures and the use of humor. Therefore, the direct physiological basis of humor style preference remains unknown. To address this issue, the present study pioneered the investigation of humor style prediction from the global and local efficiencies of brain networks while considering and controlling for the influence of gender, age, educational level, and the Big Five personality traits (Greengross et al., 2011; Liu, 2012; Wu et al., 2016) to explain the relationship between neural connections and mental health. Based on the aforementioned references on the relationship between humor style and personality (Martin et al., 2003; Liu, 2012), as well as the link between personality and brain WM (Hoptman et al., 2002; Gurrera et al., 2007; Xu and Potenza, 2012; Bjørnebekk et al., 2013), the present study hypothesized that the connectivity of WM would have a positive correlation with positive humor styles but a negative correlation with negative humor styles. Additionally, in accordance with previous brain imaging research (Singer and Lamm, 2009; Xu and Potenza, 2012), the present study hypothesized that kind-hearted humor, which is related to empathy and the self-regulation of emotions, would be positively correlated with the nodal efficiency of the temporal lobe, PFC, and posterior cingulate gyrus; hostile humor, which is related to aggressive tendencies, would be positively correlated with the nodal efficiency of the anterior cingulate gyrus and PFC; and in general, humor styles related to self-awareness would have a positive connection to the nodal efficiency of the left hemisphere.

## Materials and Methods

### Participants

Total thirty-three neurologically were recruited and collected the MRI data. Systematic visual inspection of the raw images was first executed to check the imaging quantity and 1 subject were excluded because of uncovering the whole brain. All the other subjects’ raw images were acceptable by visual experience (including motion and other distortion/artifacts). Besides, two subjects were excluded because of different nationality (*n* = 1) and missing the behavior scores (*n* = 1). Finally, thirty neurologically healthy volunteers (17 females; 24.72 ± 2.52 years old, range: 21–30 years old; 16.03 ± 0.96 years of education, range: 14–18 years) were included in this study. All of the participants were recruited from the campus in Taiwan and did not have a history of neurological or psychiatric disorders. Participants were asked to refrain from ingesting caffeine and alcohol for the 24 h preceding the experiment. The study was approved by the Research Ethics Committee of National Taiwan University Hospital. All subjects gave their informed consent to participate before commencing the study.

### Humor Style Questionnaire

The humor style questionnaire, which contained 32 items, was used to assess the four styles of humor: affiliative, self-enhancing, aggressive, and self-defeating (Chan et al., 2011). Each style was measured by eight items and rated on a seven-point scale. The higher the score was, the stronger the tendency of humor style. The internal consistencies of each style were 0.88, 0.82, 0.73, and 0.77, respectively. The criterion-related validity was found by taking the scores of personality, aggressive behavior, and self-esteem assessments as criteria: the positive humor styles (e.g., affiliative and self-enhancing) were positively related to a positive personality (e.g., openness, extraversion, and agreeableness) and self-esteem, while the negative humor styles (e.g., aggressive and self-defeating) were negatively related to a positive personality and positively related to aggressive behavior.

### MRI Acquisition

Images were acquired with a 3T scanner (Siemens Trio, Siemens Medical Solutions USA, Inc., Malvern, PA, United States) at National Taiwan University Hospital, Taiwan. Diffusion MRI data were acquired using a single-shot echo planar imaging-based sequence with sensitivity encoding and a parallel imaging factor of 2.0 and the following parameters: coverage of the whole brain; 2.5 mm slice thickness with no interslice gap; 60 axial slices; repetition time (TR) = 11000 ms; echo time (TE) = 98 ms; 30 optimal non-linear diffusion weighting directions with b = 1000 s/mm^2^ and one additional images without diffusion weighting (i.e., b = 0 s/mm^2^); average = 3; acquisition matrix = 256 × 256; field of view (FOV) = 256 mm × 256 mm, 1 mm × 1 mm × 1 mm resolution. A T1-weighted MPRAGE sequence was used to acquire high-resolution anatomical images of the entire brain with the following parameters: TR = 1560 ms, TE = 3.68 ms, flip angle = 15°, FOV = 256 mm × 256 mm, matrix size = 256 × 256; 192 sagittal slices; 1 mm × 1 mm × 1 mm resolution.

### Data Preprocessing

The preprocessing pipeline for each subject was composed of the following steps: stripping of skull and other non-cerebral material from both the T1-weighted image and dMRIs, correcting eddy currents and movements by the EDDY tool with replacing the outliers which can estimate the mean framewise dispacement and slice outliers of the data (Jesper and Stamatios, 2016; Jesper et al., 2016), fitting and eigen-decomposition of diffusion tensor, and computation of FA volume.

### Construction of Binary White Matter Connectivity Networks

All of the network construction was implemented by PANDA (Cui et al., 2013). The detailed definitions of the node and the edge are described below.

#### Network Node Definition

In this study, the automated anatomical labeling (AAL) atlas was used to segment the cerebral cortex of each subject into 1024 regions (512 for each hemisphere) and did not include the cerebellum (Hagmann et al., 2008; van den Heuvel et al., 2008; Bai et al., 2012). Each region represents a node of the DTI-based WM network. The detailed parcellating processes were implemented according to the procedure proposed by Gong et al. (2009). Briefly, the T1-weighted image was first non-linearly normalized to Montreal Neurological Institute (MNI) space by FMRIB’s Linear Image Registration Tool (FNIRT, FSL^1^). Second, the FA image of each subject was coregistered to the individual T1-weighted image. Finally, to transform the atlas from MNI space to DTI diffusion native space, the inverse transformations from the above two steps were applied to the atlas.

#### Network Edge Definition

In this study, the deterministic fiber assignment continuous tracking (FACT) algorithm was applied to reconstruct whole-brain tracts (Mori et al., 1999) by the Diffusion Toolkit^2^ which is embedded in PANDA. Specifically, the tracking procedure terminated if the turn angle of the fiber was greater than 45° or the fiber entered a voxel with a FA of less than 0.2. A two-region pair, A and B, was considered to be structurally connected if there existed at least three tracts with terminal points existing in regions A and B. Combining the definition of the nodes and the edge, a 1024 × 1024 binary network, whose elements only indicated the existence/absence of the edge between any pairwise regions, was obtained for each subject.

### Graph Theoretical Approaches

Graph theoretical measures were used to characterize topological architectures of the WM brain networks derived above. In the current study, both global network metrics and nodal metrics were computed. The global metrics of the network were computed for the mean clustering coefficient (*Cp*), characteristic path length (*Lp*), global efficiency (*E_glob_*), and local efficiency (*E_loc_*). The nodal metric of the network was computed for the nodal efficiency (*E_nodal_*).

#### Clustering Coefficient (*Cp*)

The clustering coefficient of a network characterized the segregation ability of the network with the definition of the global mean of the clustering coefficient over all nodes, whereas the clustering coefficient of a node was defined as the ratio of the number of existing connections among the node’s neighbors over all of their possible connections.

#### Characteristic Path Length (*Lp*)

The characteristic path length was used to characterize the optimal routing for information transfer. The characteristic path length of a graph refers to the averaged shortest path lengths across all nodes, where shortest path length of a node, i, was computed as the average number of distinct edges along the shortest path between node i and all other nodes in the networks. The characteristic path length of a network was computed as follows: *L_p_* = $\frac{1}{N(N-1)}$ ∑_*i*∈*G*_ ∑_*j*∈*G*_ $\frac{1}{L_{\mathrm{ij}}}$, where N is the number of nodes in the graph G, and *L_ij_* is the shortest path length between nodes i and j.

#### Global Efficiency (*E_glob_*)

Global efficiency is a global measure of the parallel information transfer ability of the whole network. It is computed as the average of the inverse of the “harmonic mean” of the characteristic path length as follows (Latora and Marchiori, 2001): *E_glob_* = $\frac{1}{N(N-1)}$ ∑_*i*≠*j*∈*G*_ $\frac{1}{L_{\mathrm{ij}}}$, where N is the number of nodes in the graph G, and *L_ij_* is the shortest path length between nodes i and j.

#### Local Efficiency (E_*loc*_)

Local efficiency quantifies the network’s ability to tolerate faults, corresponding to the efficiency of the information flow between the nearest neighbors of the node i (cf). The local efficiency of a network is computed as follows: *E_loc_* = $\frac{1}{N}$ ∑_*i*∈*G*_*E_glob_*(*G_i_*), where G_*i*_ is the subgraph composed of the nearest neighbors of node i and the connections between them.

#### Nodal Efficiency (E_*nodal*_)

Nodal efficiency is a measure of the nodal capacity to communicate with other nodes of the network. The nodal efficiency for a given node (*E_nodal_*) was defined as the inverse of the harmonic mean of the shortest path length between this node and all other nodes in the network and is computed as follows (Achard and Bullmore, 2007): *E_nodal_*(*i*) = $\frac{1}{N-1}$ ∑_*i*≠*j*∈*G*_ $\frac{1}{L_{\mathrm{ij}}}$, where *L_ij_* is the characteristic path length between node *i* and node *j*.

### Statistical Analysis

Initially, analyses of the differences and relationships between four kinds of humor styles via repeated-measure analysis of variance (ANOVA) and Pearson correlation analysis were conducted. Subsequently, to explore the correlation between the topological parameters (*Cp, Lp,* *E_loc_, E_glob,_* and *E_nodal_*) of WM brain networks and humor style scores, general linear models (GLMs) were applied, with age, gender, years of education, and the big five personality traits as covariates. Specifically, the GLM are as follows: Y = β_0_ + β_1_ × X + β_2_ × Age + β_3_ × Gender + β_4_ × Education Years + β_5_ × openness + β_6_ × extraversion + β_7_ × agreeableness + β_8_ × conscientiousness + β_9_ × neuroticism, where X is the topological parameter and Y is the different humor style score. The correlation was determined by examining the null hypothesis of β_1_ = 0. Because this was the first exploratory study that investigated the association between WM connectivity and humor styles, the threshold value for establishing the significance of correlation was set at *p* < 0.05 for the global metrics and uncorrected *p* < 0.005 for the nodal metrics of the AAL-based networks, which included 1024 multiple comparisons.

## Results

### Behavioral Results

**Table 1** lists the statistical analysis of the humor styles, including the mean, SD, range, and intercorrelation of scores. The repeated measure ANOVA results show that the difference in the tendency of the four humor styles was significant [*F*(3,87) = 53.20, *p* < 0.001, *η*^2^ = 0.65]; individuals had the highest tendency for the affiliative humor style (*M* = 5.30, *SD* = 0.79) followed by the self-enhancing humor style (*M* = 4.58, *SD* = 0.73), the self-defeating humor style (*M* = 3.94, *SD* = 0.83), and the aggressive humor style (*M* = 3.12, *SD* = 0.72). The results show that individuals preferred friendly humor styles with good intentions and tended to use hostile humor with interpersonal tension less often. In contrast, after controlling for the influence of gender, age, and educational level, the correlation of affiliative humor and self-enhancing humor (*r* = 0.43, *p* = 0.024), as well as the correlation of aggressive humor and self-defeating humor (*r* = 0.45, *p* = 0.019), were significant.

**Table 1:** Descriptive data and inter-correlations of scores on humor styles.

	Mean ± SD	Range	1	2	3	4
(1) Affiliative humor	5.30 ± 0.79	3.50–6.75	–			
(2) Self-enhancing humor	4.58 ± 0.73	2.63–5.50	0.43^∗^	–		
(3) Aggressive humor	3.12 ± 0.72	1.75–4.88	0.08	0.02	–	
(4) Self-defeating humor	3.94 ± 0.83	2.63–5.38	0.10	0.17	0.45^∗^	–

*^∗^p < 0.05.*

### Humor Styles and Network Efficiencies

Self-enhancing humor had a positive correlation with clustering coefficients (*r* = 0.46, *p* = 0.031); aggressive humor was negatively correlated with clustering coefficients (*r* = xyv0.43, *p* = 0.043), local efficiency (*r* = −0.46, *p* = 0.032), and global efficiency (*r* = −0.51, *p* = 0.015) and positively correlated with characteristic path length (*r* = 0.50, *p* = 0.017); and affiliative humor and self-defeating humor were not related to nodal efficiency. **Table 2** lists the result of the GLM by taking gender, age, educational level, and the big five personality traits as control variables.

**Table 2:** Partial correlation coefficients between network metrics and humor style (*N* = 30).

	Affiliative humor	Self-enhancing humor	Aggressive humor	Self-defeating humor
Clustering coefficient	0.01	0.46^∗^	−0.43^∗^	0.03
Characteristic path length	−0.07	−0.26	0.50^∗^	0.10
Local efficiency	−0.09	0.32	−0.46^∗^	−0.17
Global efficiency	0.03	0.21	−0.51^∗^	−0.19

*The partial correlations were computed using age, gender, education years, and big five personality as confounding covariates. ^∗^p < 0.05.*

**FIGURE 1:**
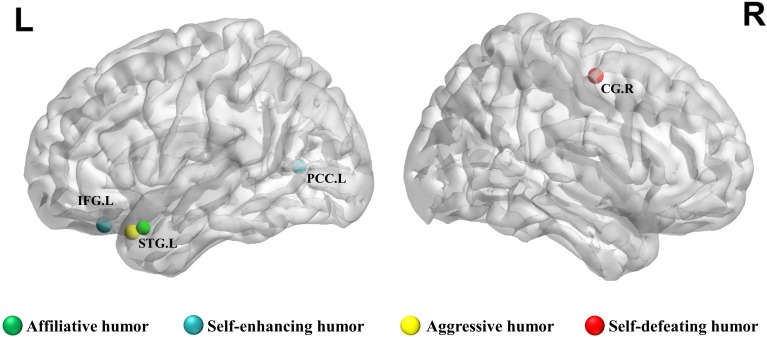
The spatial distribution of cortical regions showing a significant relationship between nodal efficiency and humor styles score (*p* < 0.005). R, right hemisphere; L, left hemisphere; CG, cingulate gyrus; IFG, inferior frontal gyrus; PCC, posterior cingulate; STG, superior temporal gyrus.

**Table 3** lists the relationship between the nodal efficiency of brain network metrics and humor styles, and the relationship is illustrated in the 3D graphs in **Figure 1**. The results show that after controlling for the influence of gender, age, educational level, and the big five personality traits, affiliative humor had positive correlations with the nodal efficiency of the left superior temporal gyrus (*r* = 0.60, *p* = 0.003); self-enhancing humor had positive correlations with the nodal efficiency of the left inferior frontal gyrus (*r* = 0.73, *p* < 0.001) and posterior cingulate gyrus (*r* = 0.65, *p* = 0.001); aggressive humor had a negative correlation with the left superior temporal gyrus (*r* = −0.68, *p* < 0.001); and self-defeating humor had a positive correlation with the nodal efficiency of the right cingulate gyrus (*r* = 0.69, *p* < 0.001).

**Table 3:** Cortical regions whose nodal efficiency significantly correlated to humor styles.

Region	X	Y	Z	*R*	*p*
**Affiliative humor**					
Left superior temporal gyrus	−45	13	−24	0.60	0.003
**Self-enhancing humor**					
Left inferior frontal gyrus	−14	32	−23	0.73	<0.001
Left posterior cingulate	0	−66	7	0.65	0.001
**Aggressive humor**					
Left superior temporal gyrus	−37	18	−27	−0.68	<0.001
**Self-defeating humor**					
Right cingulate gyrus	6	−6	52	0.69	<0.001

## Discussion

The present study investigated the preliminary relationship between specific humor styles and the connectivity efficiency of brain WM network structures by pioneering the use of graph theoretical analysis and correlating brain regional nodal efficiency with humor styles to determine how neural cognitive factors influence individual humor styles. The results show that the clustering coefficients of brain WM were positively linked to self-enhancing humor but negatively linked to aggressive humor. Regarding brain regional efficiency, affiliative humor was positively correlated with nodal efficiency of the left superior temporal gyrus. Self-enhancing humor had positive correlations with the connectivity efficiencies of the left inferior frontal gyrus and posterior cingulate gyrus. Aggressive humor was negatively correlated to the nodal efficiency of the left superior temporal gyrus. Self-defeating humor was positively correlated to the nodal efficiency of the right cingulate gyrus. In sum, the corresponding brain regions of each humor style rarely overlapped, indicating that each brain region has its own influence on the corresponding humor style.

### Topological Organization in the WM Networks and Humor Styles

The structures of brain tissue and neural fibers are fine, and the connection of brain regions forms a kind of network (Sporns et al., 2004; Gong et al., 2009). The present study used graph theory to analyze the connectivity efficiency of nodes in WM networks (Bullmore and Sporns, 2009; He et al., 2009), and the results indicated that the clustering coefficients of WM networks could positively predict the use of self-enhancing humor and were negatively connected to aggressive humor. The clustering coefficient evaluates the density of a connection between a node and its surrounding nodes in a network, as well as the efficiency of communication between nodes of regional networks (Sporns et al., 2004). Therefore, with a better regional connection, more self-encouragement with humor was used and less hostile humor was used. Self-enhancing humor had a positive correlation with subjective well-being and happiness (Martin et al., 2003; Liu, 2012), suggesting that individuals have stronger mental health when they have a sense of humor. Moreover, aggressive humor had a positive correlation with neuroticism and the tendency to attack and react with hostility (Martin et al., 2003), suggesting that communicating to others with aggressive humor might reflect a particular psychological status. Previous research of structural brain images also found that the connectivity efficiency of people with major depression or schizophrenia was not better than those without major depression or schizophrenia (Hoptman et al., 2002; Liu et al., 2008; Zhang et al., 2011; Bai et al., 2012). In sum, people with better brain regional connectivity efficiency use humor as a way to handle life and consequently have a preferable mental status.

However, the results of the present study indicate that brain connectivity efficiency had no significant correlation with affiliative humor or self-defeating humor. Affiliative humor and self-defeating humor are examples of social skills used among the four humor styles (Yip and Martin, 2006); instead of being a way to maintain psychological health, witted teasing or self-degradation is helpful in interpersonal situations (Martin et al., 2003; Chan et al., 2011), which may be the reason for the lack of a direct relationship between connectivity efficiency and affiliative and self-defeating humor.

### Affiliative Humor

Affiliative humor had a positive correlation with the nodal efficiency of the left superior temporal gyrus. Affiliative humor is where individuals please others or decrease interpersonal conflict by using friendly and interesting ways to solve the dilemma of social interaction (Martin et al., 2003; Chan et al., 2011). Affiliative humor is highly related to theory of mind (Wu et al., 2014), empathy (Hampes, 2010), self-esteem (Stieger et al., 2011), openness and extraverted personalities (Chan et al., 2011). According to the results of the present study, the brain regions that had significant connections were all involved with the aforementioned psychological processes; the superior temporal gyrus and openness were positively correlated (Kitamura et al., 2016). Our results support past findings of behavioral studies showing that affiliative humor and other psychological traits are related. Additionally, the consistency of our findings with those of other brain imaging studies supports the hypothesis that specific brain regions have an influence on the tendency to use affiliative humor.

### Self-Enhancing Humor

Self-enhancing humor had a significant positive correlation with the nodal efficiency of the left inferior temporal gyrus and posterior cingulate gyrus. Self-enhancing humor is where individuals use positive and humorous ways to cope with the pressure of life and difficulties. The concept is close to humor in the narrow sense and exhibits positive correlations to openness, agreeableness, extraversion (Martin et al., 2003; Chan et al., 2011), and empathy (Hampes, 2010) but a negative correlation to neuroticism. It is an ideal psychological state. Past brain imaging research has indicated that the aforementioned brain regions had a significant relationship with self-awareness (cingulate gyrus), emotion recognition (inferior frontal gyrus), empathy (inferior frontal gyrus, posterior cingulate gyrus), and positive personality (i.e., openness, extraversion, agreeableness) (posterior cingulate gyrus) (Carr et al., 2003; Maddock et al., 2003; Rizzolatti and Craighero, 2004; Kumari et al., 2007; Hooker et al., 2008; Kunisato et al., 2011; Schurz et al., 2014). Our results are consistent with the behavioral findings; with the support of brain imaging data, they suggest that the operation of the aforementioned brain regions had a positive influence on the tendency toward self-enhancing humor, resulting in better physical and psychological health.

According to the results of the present study, self-enhancing humor was significantly linked to nodes in the left cerebral hemisphere, such as the inferior temporal gyrus and posterior cingulate gyrus. These results agreed with the hypothesis that the left cerebral hemisphere was important for processing information about the self and others (Turk et al., 2002; Denny et al., 2012). Moreover, past research has indicated that efficient operation in the parietal and temporal lobes had an effect on the positive psychological traits of individuals, such as empathy, openness, extraversion, and agreeableness (Knutson et al., 2003, 2005; Ochsner et al., 2004). These results support the idea that individuals would have better mental health if they tended to use some positive ways to cope with the pressures of life, such as self-encouragement (Martin et al., 2003; Chan et al., 2011), and they indicate that among the four humor styles, self-enhancing humor is a good style for a healthy psychological status.

### Aggressive Humor

Aggressive humor had a negative correlation to the left superior temporal gyrus. Aggressive humor means that an individual enhances self by way of mocking others, such as scorning, ridiculing, digging, and sneering at others. Certain hostile expressions led to interpersonal tension and had a positive correlation with neuroticism, hostility, and the tendency to attack (Martin et al., 2003; Chan et al., 2011) but a negative correlation with empathy (Hampes, 2010). Past brain imaging studies have shown that the superior temporal gyrus had a positive connection to openness (Kitamura et al., 2016), whereas aggressive humor had a negative relationship with openness (Chan et al., 2011). These findings agree with our finding that the nodal efficiency of the superior temporal gyrus and aggressive humor had a negative correlation. Interestingly, our results are not similar to the findings on affiliative humor; they suggest that the connectivity efficiency of the left superior temporal gyrus is an important neurocognitive factor in the decision of whether to use affiliative or aggressive humor.

### Self-Defeating Humor

Self-defeating humor was significantly positively linked to the connectivity efficiency of the right cingulate gyrus. Self-defeating humor increases interpersonal relationships by way of abusing oneself and amusing others, which means making others happy by downgrading self-esteem, with the intention of obtaining the attention or recognition of others even when it hurts deeply inside. Self-defeating humor is positively correlated to neuroticism but negatively correlated to self-esteem and subjective well-being (Martin et al., 2003; Chan et al., 2011; Stieger et al., 2011). Self-defeating humor and the nodal efficiency of the cingulate gyrus had a positive correlation. This result is consistent with the finding in self-enhancing humor. In sum, the cingulate gyrus was relevant to self-awareness (Schurz et al., 2014); both self-defeating and self-enhancing humor styles focused on the evaluation of the internal self-status (Martin et al., 2003), and the brain structure imaging results support this theory.

### Limitations/Future Studies

The present study is groundbreaking but is likely underpowered to explore the connection between humor styles and brain WM. Due to our limited research budget, we used only thirty subjects, which is a small-scale sample size and still poses a dilemma for personality neuroscience research (Abram and DeYoung, 2017). However, the present study controlled for the influence of related variables, including gender, age, years of education, and the big five personality traits, and we also present valuable results and inspiration for further research in spite of the small sample. Additionally, humor has many aspects, such as cognition, disposition, and application (Wu et al., 2014), and the humor process has different paths for the development of incongruity-resolution humor and nonsense humor (Samson et al., 2008) and how individuals automatically develop the ability to create humor (Amir and Biederman, 2016). Simultaneously, humor styles have been found to be significantly correlated with verbal intelligence (Greengross et al., 2011). The present study only focused on how humor was used. In future studies, it would be worthwhile to investigate WM structure and its correspondence with the ability to understand different kinds of humor, as well as humor creation and humor preference after controlling for verbal intelligence. Regarding the brain imaging technique, the present study used a 30-direction diffusion sequence; to obtain better-quality images, we suggest using a 64-direction diffusion sequence as well as precisely collecting and correcting for electric field intensity and head motion in the future. Finally, researchers have started to use typological approaches to describe the relationship between humor styles and other psychological traits in recent years. In these studies, cluster analysis was used for the classification of people with the tendency to use different humor styles (Galloway, 2010; Chang et al., 2015), such as general humor endorser, humor denier, positive humor endorser, and negative humor endorser; and then differences in specific psychological traits (e.g., creativity and personality) in each type are compared. However, these research methods have a sample size requirement. For future research, the number of participants should be increased, thus allowing for the examination of differences in WM structure across every category of humor tendency.

## Conclusion

The present study finds that the efficiency of WM regional communication predicts a positive correlation with self-enhancing humor and a negative correlation with aggressive humor. Moreover, the nodal efficiency of the left superior temporal gyrus could help individuals judge a situation and cope with social difficulties in either a friendly or hostile way. These results support the hypothesis that WM structure has a critical influence on the use of certain humor styles, as well as that the technique of DTI helps to assess the status of individual psychological health and the use of humor.

## Author Contributions

C-LW and SZ collected and analyzed the data and wrote the initial draft of the manuscript. C-LW assisted in literature review and discussion. H-CC designed this study. YH and Y-CC monitored and supervised all aspects of the study. All authors approved the final version of the paper.

## Conflict of Interest Statement

The authors declare that the research was conducted in the absence of any commercial or financial relationships that could be construed as a potential conflict of interest.

## Footnotes

1 http://www.fmrib.ox.ac.uk/fsl/

2 http://trackvis.org

## References

[B1] Abram S. V. DeYoung C. G. (2017). Using personality neuroscience to study personality disorder. *Pers. Disord.* 8 2–13. 10.1037/per0000195 28045302

[B2] Achard S. Bullmore E. (2007). Efficiency and cost of economical brain functional networks. *PLoS Comput. Biol.* 3:e17. 10.1371/journal.pcbi.0030017 17274684 PMC1794324

[B3] Achard S. Salvador R. Whitcher B. Suckling J. Bullmore E. (2006). A resilient, low-frequency, small-world human brain functional network with highly connected association cortical hubs. *J. Neurosci.* 26 63–72. 10.1523/JNEUROSCI.3874-05.2006 16399673 PMC6674299

[B4] Amir O. Biederman I. (2016). The neural correlates of humor creativity. *Front. Hum. Neurosci.* 10:597. 10.3389/fnhum.2016.00597 27932965 PMC5122582

[B5] Bai F. Shu N. Yuan Y. Shi Y. Yu H. Wu D. (2012). Topologically convergent and divergent structural connectivity patterns between patients with remitted geriatric depression and amnestic mild cognitive impairment. *J. Neurosci.* 32 4307–4318. 10.1523/JNEUROSCI.5061-11.2012 22442092 PMC6621223

[B6] Bjørnebekk A. Fjell A. M. Walhovd K. B. Grydeland H. Torgersen S. Westlye L. T. (2013). Neuronal correlates of the five factor model (FFM) of human personality: multimodal imaging in a large healthy sample. *Neuroimage* 65 194–208. 10.1016/j.neuroimage.2012.10.009 23063449

[B7] Bullmore E. Sporns O. (2009). Complex brain networks: graph theoretical analysis of structural and functional systems. *Nat. Rev. Neurosci.* 10 186–198. 10.1038/nrn2575 19190637

[B8] Carr L. Iacoboni M. Dubeau M. C. Mazziotta J. C. Lenzi G. L. (2003). Neural mechanisms of empathy in humans: a relay from neural systems for imitation to limbic areas. *Proc. Natl. Acad. Sci. U.S.A.* 100 5497–5502. 10.1073/pnas.0935845100 12682281 PMC154373

[B9] Chan Y. C. Chen H. C. Cho S. L. Martin R. A. (2011). Distingguishing between kindhearted and malicious humor: development of a traditional chinese version of the humor styles questionnaire. *Psychol. Test.* 207–234.

[B10] Chan Y. C. Chou T. L. Chen H. C. Liang K. C. (2012). Segregating the comprehension and elaboration processing of verbal jokes: an fMRI study. *Neuroimage* 61 899–906. 10.1016/j.neuroimage.2012.03.052 22472220

[B11] Chan Y. C. Chou T. L. Chen H. C. Yeh Y. C. Lavallee J. P. Liang K. C. (2013). Towards a neural circuit model of verbal humor processing: an fMRI study of the neural substrates of incongruity detection and resolution. *Neuroimage* 66 169–176. 10.1016/j.neuroimage.2012.10.019 23103517

[B12] Chang J.-H. Chen H.-C. Hsu C.-C. Chan Y.-C. Chang Y.-L. (2015). Flexible humor styles and the creative mind: using a typological approach to investigate the relationship between humor styles and creativity. *Psychol. Aesthet. Creat. Arts* 9 306–312. 10.1037/a0039527

[B13] Cui Z. Zhong S. Xu P. He Y. Gong G. (2013). PANDA: a pipeline toolbox for analyzing brain diffusion images. *Front. Hum. Neurosci.* 21:42. 10.3389/fnhum.2013.00042 23439846 PMC3578208

[B14] Denny B. T. Kober H. Wager T. D. Ochsner K. N. (2012). A meta-analysis of functional neuroimaging studies of self- and other judgments reveals a spatial gradient for mentalizing in medial prefrontal cortex. *J. Cogn. Neurosci.* 24 1742–1752. 10.1162/jocn_a_00233 22452556 PMC3806720

[B15] Denson T. F. Pedersen W. C. Ronquillo J. Nandy A. S. (2009). The angry brain: neural correlates of anger, angry rumination, and aggressive personality. *J. Cogn. Neurosci.* 21 734–744. 10.1162/jocn.2009.21051 18578600

[B16] DeYoung C. G. Hirsh J. B. Shane M. S. Papademetris X. Rajeevan N. Gray J. R. (2010). Testing predictions from personality neuroscience. *Psychol. Sci.* 21 820–828. 10.1177/0956797610370159 20435951 PMC3049165

[B17] Dozois D. J. A. Martin R. A. Bieling P. J. (2009). Early maladaptive schemas and adaptive/maladaptive styles of humor. *Cogn. Ther. Res.* 33 585–596. 10.1007/s10608-008-9223-9

[B18] Farrow T. F. Zheng Y. Wilkinson I. D. Spence S. A. Deakin J. F. Tarrier N. (2001). Investigating the functional anatomy of empathy and forgiveness. *Neuroreport* 12 2433–2438. 10.1097/00001756-200108080-00029 11496124

[B19] Galloway G. (2010). Individual differences in personal humor styles: identification of prominent patterns and their associates. *Pers. Individ. Diff.* 48 563–567. 10.1016/j.paid.2009.12.007

[B20] Goel V. Dolan R. J. (2001). The functional anatomy of humor: segregating cognitive and affective components. *Nat. Neurosci.* 4 237–238. 10.1038/85076 11224538

[B21] Gong G. Rosa-Neto P. Carbonell F. Chen Z. J. He Y. Evans A. C. (2009). Ageand gender-related differences in the cortical anatomical network. *J. Neurosci.* 29 15684–15693. 10.1523/JNEUROSCI.2308-09.2009 20016083 PMC2831804

[B22] Greengross G. Martin R. A. Miller G. (2011). Personality traits, intelligence, humor styles, and humor production ability of professional stand-up comedians compared to college students. *Psychol. Aesthet. Creat. Arts* 6 74–82. 10.1037/a0025774

[B23] Gurrera R. J. Nakamura M. Kubicki M. Dickey C. C. Niznikiewicz M. A. Voglmaier M. M. (2007). The uncinated fasciculus and extraversion in schizotypal personality dis-order: a diffusion tensor imaging study. *Schizophrenia Res.* 90 360–362. 10.1016/j.schres.2006.10.003 17126532 PMC1876710

[B24] Haas B. W. Omura K. Amin Z. Constable R. T. Canli T. (2006). Functional connectivity with the anterior cingulate is associated with extraversion during the emotional Stroop task. *Soc. Neurosci.* 1 16–24. 10.1080/17470910600650753 18633773

[B25] Hagmann P. Cammoun L. Gigandet X. Meuli R. Honey C. J. Wedeen V. J. (2008). Mapping the structural core of human cerebral cortex. *PLoS Biol.* 6:e159. 10.1371/journal.pbio.0060159 18597554 PMC2443193

[B26] Hampes W. P. (2001). Relation between humor and empathic concern. *Psychol. Rep.* 88 241–244. 10.2466/pr0.2001.88.1.241 11293036

[B27] Hampes W. P. (2010). The relation between humor styles and empathy. *Eur. J. Psychol.* 6 34–45. 10.5964/ejop.v6i3.207

[B28] Harenski C. L. Sang H. K. Hamann S. (2009). Neuroticism and psychopathy predict brain activation during moral and nonmoral emotion regulation. *Cogn. Affect. Behav. Neurosci* 9 1–15. 10.3758/CABN.9.1.1 19246323

[B29] He Y. Chen Z. J. Gong G. Evans A. C. (2009). Neuronal networks in Alzheimer’s disease. *Neuroscientist* 15 333–350. 10.1177/1073858409334423 19458383

[B30] Hooker C. I. Verosky S. C. Germine L. T. Knight R. T. D’Esposito M. (2008). Mentalizing about emotion and its relationship to empathy. *Soc. Cogn. Affect. Neurosci.* 3 204–217. 10.1093/scan/nsn019 19015112 PMC2566770

[B31] Hoptman M. J. Volavka J. Johnson G. Weiss E. Bilder R. M. Lim K. O. (2002). Frontal white matter microstructure, aggression, and impulsivity in men with schizophrenia: a preliminary study. *Biol. Psychiatry* 52 9–14. 10.1016/S0006-3223(02)01311-2 12079725

[B32] Jesper L. R. A. Mark S. G. Eniko Z. Stamatios N. S. (2016). Incorporating outlier detection and replacement into a non-parametric framework for movement and distortion correction of diffusion MR images. *Neuroimage* 141 556–572. 10.1016/j.neuroimage.2016.06.058 27393418

[B33] Jesper L. R. A. Stamatios N. S. (2016). An integrated approach to correction for off-resonance effects and subject movement in diffusion MR imaging. *Neuroimage* 125 1063–1078. 10.1016/j.neuroimage.2015.10.019 26481672 PMC4692656

[B34] Kitamura S. Yasuno F. Yamamoto A. Kazui H. Kudo T. Matsuoka K. (2016). A structural model of age, grey matter volumes, education, and personality traits. *Psychogeriatrics* 16 46–53. 10.1111/psyg.12118 25735496

[B35] Knutson B. Fong G. W. Bennett S. M. Adams C. M. Hommer D. (2003). A region of mesial prefrontal cortex tracks monetarily rewarding outcomes: characterization with rapid event-related fMRI. *Neuroimage* 18 263–272. 10.1016/S1053-8119(02)00057-5 12595181

[B36] Knutson B. Taylor J. Kaufman M. Peterson R. Glover G. (2005). Distributed neural representation of expected value. *J. Neurosci.* 25 4806–4812. 10.1523/JNEUROSCI.0642-05.2005 15888656 PMC6724773

[B37] Kuhl J. Kazén M. (2008). Motivation, affect, and hemispheric asymmetry: power versus affiliation. *J. Pers. Soc. Psychol.* 95:456. 10.1037/0022-3514.95.2.456 18665713

[B38] Kuiper N. A. Grimshaw M. Leite C. Kirsh G. (2004). Humor is not always the best medicine: specific components of sense of humor and psychological wellbeing. *Hum. Int. J. Hum. Res.* 17 135–168. 10.1515/humr.2004.002

[B39] Kumari V. Ffytche D. H. Das M. Wilson G. D. Goswami S. Sharma T. (2007). Neuroticism and brain responses to anticipatory fear. *Behav. Neurosci.* 121 643–652. 10.1037/0735-7044.121.4.643 17663590

[B40] Kunisato Y. Okamoto Y. Okada G. Aoyama S. Nishiyama Y. Onoda K. (2011). Personality traits and the amplitude of spontaneous low-frequency oscillations during resting state. *Neurosci. Lett.* 492 109–113. 10.1016/j.neulet.2011.01.067 21291958

[B41] Latora V. Marchiori M. (2001). Efficient behavior of small-world networks. *Phys. Rev. Lett.* 87 198701–198704. 10.1103/PhysRevLett.87.198701 11690461

[B42] Lefcourt H. M. (2001). *Humor: The Psychology of Living Buoyantly.* New York, NY: Kluwer Academic Publishers 10.1007/978-1-4615-4287-2

[B43] Liu K. W. Y. (2012). Humor styles, self-esteem and subjective happiness. *Discov. SS Stud. E J.* 1 21–41. 10.2466/07.02.PR0.115c18z6 25153846

[B44] Liu Y. Liang M. Zhou Y. He Y. Hao Y. Song M. (2008). Disrupted small-world networks in schizophrenia. *Brain* 131 945–961. 10.1093/brain/awn018 18299296

[B45] Long D. Graesser A. (1988). Wit and humor in discourse processing. *Discourse Process.* 11 35–60. 10.1080/01638538809544690 16815790

[B46] Maddock R. J. Garrett A. S. Buonocore M. H. (2003). Posterior cingulate cortex activation by emotional words: fMRI evidence from a valence decision task. *Hum. Brain Mapp.* 18 30–41. 10.1002/hbm.10075 12454910 PMC6871991

[B47] Mahy C. E. V. Moses L. J. Pfeifer J. H. (2014). How and where: theory-of-mind in the brain. *Dev. Cogn. Neurosci.* 9 68–81. 10.1016/j.dcn.2014.01.002 24552989 PMC6989753

[B48] Martin R. A. (2001). Humor, laughter, and physical health: methodological issues and research findings. *Psychol. Bull.* 127 504–519. 10.1037/0033-2909.127.4.504 11439709

[B49] Martin R. A. Puhlik-Doris P. Larswn G. Gray J. Weir K. (2003). Individual differences in uses of humor and their relation to psychological well-being: development of the humor styles questionnaire. *J. Res. Pers.* 37 48–75. 10.1016/S0092-6566(02)00534-2

[B50] Mobbs D. Hagan C. C. Azim E. Menon V. Reiss A. L. (2005). Personality predicts activity in reward and motional regions associated with humor. *Proc. Natl. Acad. Sci. U. S. A.* 102 16502–16506. 10.1073/pnas.0408457102 16275930 PMC1277964

[B51] Mori S. Crain B. J. Chacko V. P. van Zijl P. C. (1999). Three-dimensional tracking of axonal projections in the brain by magnetic resonance imaging. *Ann. Neurol.* 45 265–269. 10.1002/1531-8249(199902)45:2<265::AID-ANA21>3.0.CO;2-3 9989633

[B52] Ochsner K. N. Ray R. D. Cooper J. C. Robertson E. R. Chopra S. Gabrieli J. D. E. (2004). For better or for worse : neural systems supporting the cognitive down-and up-regulation negative emotion. *Neuroimage* 23 483–499. 10.1016/j.neuroimage.2004.06.030 15488398

[B53] Rizzolatti G. Craighero L. (2004). The mirror-neuron system. *Annu. Rev. Neurosci.* 27 169–192. 10.1146/annurev.neuro.27.070203.144230 15217330

[B54] Samson A. C. Huber O. Ruch W. (2013). Seven decades after Hans Asperger’s observations: a comprehensive study of humor in individuals with autism spectrum disorders. *Humor* 26 441–460. 10.1515/humor-2013-0026

[B55] Samson A. C. Zysset S. Huber O. (2008). Cognitive humor processing: different logical mechanisms in nonverbal cartoons—an fMRI study. *Soc. Neurosci.* 3 125–140. 10.1080/17470910701745858 18633854

[B56] Schurz M. Radua J. Aichhorn M. Richlana F. Perner J. (2014). Fractionating theory of mind: a meta-analysis of functional brain imaging studies. *Neurosci. Biobehav. Rev.* 42 9–34. 10.1016/j.neubiorev.2014.01.009 24486722

[B57] Singer T. Lamm C. (2009). The social neuroscience of empathy. *Ann. N. Y. Acad. Sci.* 1156 81–96. 10.1111/j.1749-6632.2009.04418.x 19338504

[B58] Sporns O. Chialvo D. R. Kaiser M. Hilgetag C. C. (2004). Organization, development and function of complex brain networks. *Trends Cogn. Sci.* 8 418–425. 10.1016/j.tics.2004.07.008 15350243

[B59] Sporns O. Tononi G. Kötter R. (2005). The human connectome: a structural description of the human brain. *PLoS Comput. Biol.* 1:e42. 10.1371/journal.pcbi.0010042 16201007 PMC1239902

[B60] Stieger S. Formann A. K. Burger C. (2011). Humor styles and their relationship to explicit and implicit self-esteem. *Pers. Individ. Diff.* 50 747–750. 10.1016/j.paid.2010.11.025

[B61] Suls J. M. (1972). “A two-stage model for the appreciation of jokes and cartoons: an information-processing analysis,” in *The Psychology of Humor: Theoretical Perspectives and Empirical Issues*, eds Goldstein J. H. McGhee P. E. (New York, NY: Academic Press), 81–100.

[B62] Takeuchi H. Taki Y. Sassa Y. Hashizume H. Sekiguchi A. Fukushima A. (2010). White matter structures associated with creativity: evidence from diffusion tensor imaging. *Neuroimage* 51 11–18. 10.1016/j.neuroimage.2010.02.035 20171286

[B63] Turk D. J. Heatherton T. F. Kelley W. M. Funnell M. G. Gazzaniga M. S. Macrae C. N. (2002). Mike or me? Self-recognition in a split-brain patient. *Nat. Neurosci.* 5 841–842. 10.1038/nn907 12195428

[B64] van den Heuvel M. P. Mandl R. C. W. Kahn R. S. Hulshoff Pol H. E. (2009). Functionally linked resting-state networks reflect the underlying structural connectivity architecture of the human brain. *Hum. Brain Mapp* 30 3127–3141. 10.1002/hbm.20737 19235882 PMC6870902

[B65] van den Heuvel M. P. Stam C. J. Boersma M. Hulshoff Pol H. E. (2008). Smallworld and scale-free organization of voxel-based resting-state functional connectivity in the human brain. *Neuroimage* 43 528–539. 10.1016/j.neuroimage.2008.08.010 18786642

[B66] Vöellm B. A. Taylor A. N. Richardson P. Corcoran R. Stirling J. McKie S. (2006). Neuronal correlates of theory of mind and empathy: a functional magnetic resonance imaging study in a nonverbal task. *Neuroimage* 29 90–98. 10.1016/j.neuroimage.2005.07.022 16122944

[B67] Wu C. L. Tseng L. P. An C. P. Chen H. C. Chan Y. C. Shih C. I. (2014). Do individuals with autism lack a sense of humor? A study of humor comprehension, appreciation, and styles among high school students with autism. *Res. Autism Spectr. Disord.* 8 1386–1393. 10.1016/j.rasd.2014.07.006

[B68] Wu C. L. Zhong S. Y. Chan Y. C. Chen H. C. Gong G. L. He Y. (2016). White-matter structural connectivity underlying human laughter-related traits processing. *Front. Psychol.* 7:1637. 10.3389/fpsyg.2016.01637 27833572 PMC5082228

[B69] Wyer R. S. Collins J. E. (1992). A theory of humor elicitation. *Psychol. Rev.* 99 663–688. 10.1037/0033-295X.99.4.663 1454903

[B70] Xu J. Potenza M. N. (2012). White matter integrity and five-factor personality measures in healthy adults. *Neuroimage* 59 800–807. 10.1016/j.neuroimage.2011.07.040 21840401 PMC3195960

[B71] Yip J. A. Martin R. A. (2006). Sense of humor, emotional intelligence, and social competence. *J. Res. Pers.* 40 1202–1208. 10.1016/j.jrp.2005.08.005

[B72] Zhang J. Wang J. Wu Q. Kuang W. Huang X. He Y. (2011). Disrupted brain connectivity networks in drug-naive, first-episode major depressive disorder. *Biol. Psychiatry* 70 334–342. 10.1016/j.biopsych.2011.05.018 21791259

